# From cognition to response: clinical challenges, antibiotic resistance mechanisms, and prevention and control strategies of *Elizabethkingia meningoseptica* infections

**DOI:** 10.3389/fmicb.2026.1774907

**Published:** 2026-05-12

**Authors:** Qinghua Zhang, Shan Cong, Yan Wang

**Affiliations:** Department of Respiratory and Critical Care Medicine, The Second Hospital of Jilin University, Changchun, Jilin, China

**Keywords:** dissemination of drug-resistant genes, *Elizabethkingia meningoseptica*, hospital-acquired infection, multidrug resistance, precision diagnosis and treatment

## Abstract

*Elizabethkingia meningoseptica* (EM), a rare but increasingly recognized Gram-negative multidrug-resistant bacterium, primarily infects neonates and immunocompromised patients, often leading to severe clinical diseases such as meningitis and sepsis. In recent years, with the increasing complexity of the medical environment, the incidence of EM infections has been on the rise, posing numerous challenges in diagnosis and treatment. This bacterium possesses complex resistance mechanisms, resulting in limited efficacy of traditional antimicrobial agents and significantly affecting patient prognosis. This article systematically reviews the epidemiological characteristics, clinical manifestations, and existing diagnostic techniques of EM, with a focus on exploring its resistance genes and mechanisms. Combining the latest genomic research findings, it analyzes the transmission routes and prevention and control difficulties of EM in the hospital environment. By integrating clinical case data, comprehensive prevention and control strategies and individualized antimicrobial treatment plans for EM infections are proposed, aiming to provide theoretical support and practical guidance for clinicians and infection control experts, and to improve the diagnosis, treatment, and prevention levels of EM infections.

## 1 Introduction

*Elizabethkingia meningoseptica* (EM), as a Gram-negative opportunistic pathogen, was first reported by Elizabeth O. King in 1959. This bacterium belongs to the Flavobacteriaceae family and is characterized by non-motility, aerobic metabolism, and being Gram-negative. It is widely distributed in natural environments such as water and soil. Meanwhile, it can also colonize and survive in medical equipment and piping systems, emerging as an important pathogen of hospital-acquired infections (Lau et al., 2016; Choi et al., 2019; Lin et al., 2019).

*Elizabethkingia meningoseptica* infections primarily affect neonates, immunosuppressed patients, and elderly patients (Raj et al., 2024; Swami et al., 2024). The most common clinical manifestations are sepsis, pneumonia, and meningitis (Waleed et al., 2021; Murthy et al., 2022; Wu et al., 2023). In the neonatal population, meningitis is the most characteristic manifestation, often complicated by sepsis or bacteremia, and may be accompanied by a series of other clinical manifestations such as osteomyelitis, urinary tract infection, endogenous endophthalmitis, endocarditis, epididymo-orchitis, lung abscess, necrotizing fasciitis, cystic fibrosis-related infections, and hydrocephalus (Jean et al., 2014; Li et al., 2022; Zajmi et al., 2022). In addition, immunocompromised adult patients, such as organ transplant recipients, cancer patients, and those on long-term bed rest or using invasive medical devices, are also prone to EM infections, and the condition is often severe (Hoa and Dang Hai, 2021; Ferreira et al., 2024), with a mortality rate of up to 57% (Ceyhan and Celik, 2011). Due to the fact that traditional colony culture and identification methods often lead to misjudgments because of similar bacterial species, especially being easily confused with *Elizabethkingia anophelis*, it causes difficulties in clinical diagnosis. With the development of molecular biology techniques and gene sequencing, accurate species identification can be achieved, which contributes to precise clinical medication and epidemiological tracking (Larkin et al., 2021; Wang et al., 2022).

The core challenge in treating EM infections is its inherent multi-drug resistance. Literature reports indicate that this bacterium exhibits resistance to most commonly used antibiotics, particularly showing widespread resistance to β-lactams and carbapenems (Kuo et al., 2021; Ma et al., 2023). Currently, antibiotics such as fluoroquinolones, sulfamethoxazole, and minocycline show relatively good sensitivity to EM, yet there is still a risk of resistance (Li et al., 2022; Alhuthil et al., 2024). Genomic studies have revealed that EM harbors various resistance genes, including multiple β-lactamase genes such as *bla*B, *bla*GOB, and *bla*CME, as well as the *tet*(X) gene. These genes promote the spread of resistance genes through integration and transferable elements, enhancing the resistance ability of EM (Morgado et al., 2024b; Cai et al., 2025). Additionally, EM can form biofilms, which increase its survival ability and the risk of infection in the hospital environment, further exacerbating the difficulties in clinical prevention and control (Li et al., 2022; Yang et al., 2024).

The persistent presence and transmission ability of EM in the hospital environment pose another challenge for prevention and control. Studies have found that EM can contaminate medical equipment such as ventilators, urinary catheters, infusion lines, and water systems, and can survive in biofilms for a long time, leading to hospital-acquired infection outbreaks through cross-contact (Ceyhan and Celik, 2011; Wang et al., 2022; Ferreira et al., 2024). Therefore, strengthening hospital infection control measures is the key to preventing EM infections.

**FIGURE 1:**
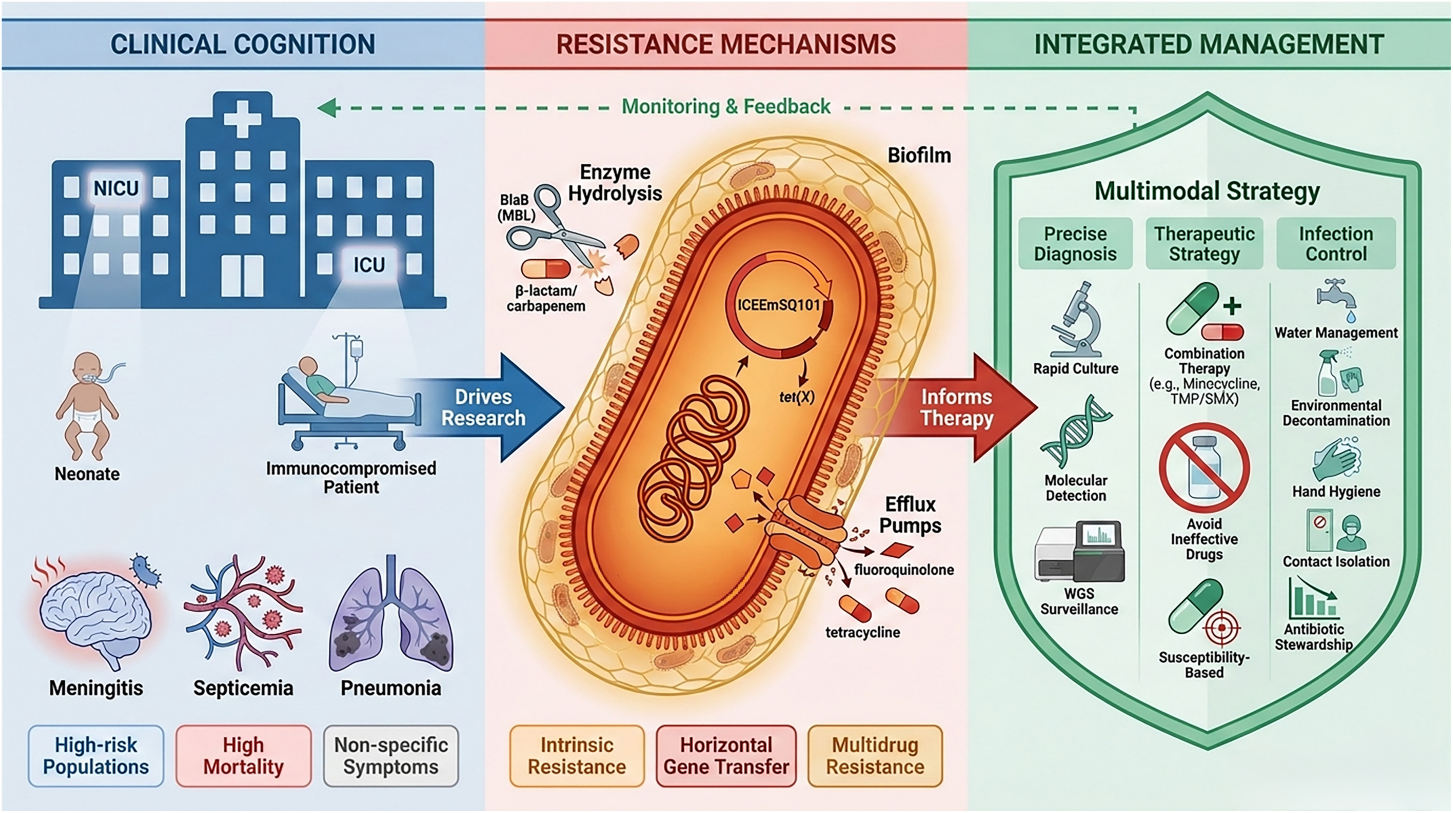
The “cognition-control” framework for combating *Elizabethkingia meningoseptica* infections. This diagram presents a systematic approach to addressing EM infections, progressing from problem identification to integrated solutions. The left panel (clinical cognition) highlights high-risk patient groups and key disease presentations. The central panel (resistance mechanisms) illustrates the molecular drivers of multidrug resistance, including intrinsic β-lactamases, efflux pumps, biofilm formation, and mobile genetic elements. The right panel (integrated management) outlines actionable strategies across three domains: rapid diagnosis, optimized therapy, and comprehensive infection control. Arrows indicate the underlying logic of research and the essential feedback loop for continuous improvement, representing a dynamic and closed-loop strategy.

Figure 1 illustrates that tackling the complex challenge of EM infection necessitates the development of a closed-loop system. This system should advance from clinical recognition to mechanistic elucidation and ultimately to integrated intervention.

In summary, EM, as a nosocomial infection pathogen with high pathogenic potential and multidrug resistance, it is of great significance to systematically summarize its epidemiological characteristics, resistance mechanisms, and prevention and control strategies. However, the current understanding in this area remains fragmented, especially in terms of clinical treatment guidelines, the transmission patterns of resistance genes, and the integration of comprehensive prevention and control strategies. This review aims to follow the framework outlined in Figure 1, systematically integrate the recent research progress and clinical data, elaborate on the clinical features and molecular resistance mechanisms of EM infections, and explore treatment strategies and prevention and control programs based on the existing evidence, with the hope of providing references for improving the clinical diagnosis, treatment, and infection control of EM infections (Ma et al., 2023; Alhuthil et al., 2024; Alsayed et al., 2025).

## 2 Epidemiology and clinical manifestation of *Elizabethkingia meningoseptica*

### 2.1 Epidemiological overview

*Elizabethkingia meningoseptica* is an opportunistic pathogen widely present in nature and hospital environments. Its epidemiological characteristics are reflected in complex environmental niches, well-defined high-risk populations, and typical hospital-acquired transmission patterns.

The ecological distribution of EM is extremely extensive, and it can exist in natural water bodies and various media in hospital environments, such as medical equipment, ventilation systems, and the surfaces of various catheters. A study on isolates from bullfrogs in Taiwan confirmed that both these environmental strains and human clinical isolates exhibit significant genetic diversity, which verifies the strong adaptability and transmission potential of EM in the environment. Using pulsed-field gel electrophoresis (PFGE) with *Apa*I restriction enzyme digestion for comparative genomic analysis, the study revealed that all 14 bullfrog isolates belonged to a single pulsotype (A6), while human isolates were classified into four distinct pulsotypes (A2–A5), with no common pulsotype shared between bullfrog and human isolates (Tsai et al., 2023). This lack of direct genetic correlation suggests that human infections may originate from the diverse strain pool in the environment rather than being directly transmitted from specific animal hosts. From an infection control perspective, this finding underscores the importance of focusing prevention efforts on environmental sources, particularly hospital water systems and medical equipment, rather than implementing measures targeting animal contact.

*Elizabethkingia meningoseptica* infection has well-defined high-risk groups. Newborns, especially premature infants, and patients with immunodeficiency are at high risk of EM infection. Neonatal infections often present as sepsis and meningitis, with a relatively high mortality rate. Numerous studies have reported that neonatal infection patients are highly susceptible to EM due to critical illness and incomplete development of the immune system, and may develop severe neurological complications such as hydrocephalus after infection (Wang et al., 2022; Swami et al., 2024). Among adult patients, immunosuppressed individuals, critically ill patients, and those with long-term use of mechanical ventilation or central venous catheters are the main affected groups. Research shows that comorbidities of underlying respiratory or cardiovascular diseases are common features in adult EM-infected patients, and receiving mechanical ventilation or immunosuppressive therapy has been identified as an independent risk factor for infection occurrence and death (Ma et al., 2023; Alhuthil et al., 2024; Alsayed et al., 2025).

*Elizabethkingia meningoseptica* infections are mostly hospital-acquired, especially with the risk of explosive nosocomial infections in the intensive care unit. Environmental monitoring has found that the water tanks of mechanical ventilation equipment in hospitals are often contaminated by EM, becoming the source of infection. Genetic analysis of nosocomial epidemic strains shows that hospital outbreaks are often caused by genetically related clonal strains, suggesting a high possibility of horizontal transmission within the hospital (Erinmez et al., 2021; Hu et al., 2023). In addition, EM has the ability to form biofilms, which enables it to stably exist on the surface of medical equipment and resist routine disinfection, and is a key factor leading to persistent or recurrent nosocomial infections (Li et al., 2022).

In summary, as an opportunistic pathogen widely distributed in the environment, the epidemiological characteristics of EM can be summarized as follows: extensive colonization in environmental media, susceptibility of specific high-risk populations, and the risk of outbreak in hospital environments, especially in intensive care units and neonatal departments. Therefore, strengthening environmental monitoring, achieving early clinical identification, and strictly implementing infection prevention and control measures are of crucial significance for blocking its in-hospital transmission and reducing the infection rate (Erinmez et al., 2021; Ma et al., 2023; Alhuthil et al., 2024; Swami et al., 2024).

### 2.2 Clinical manifestations

As an important nosocomial opportunistic pathogen, the clinical manifestations of EM vary significantly depending on the host’s immune status and age. In neonates, EM infections can lead to multi-site infections such as sepsis and pneumonia, with meningitis being the most characteristic and fatal. Infants often present symptoms such as feeding difficulties, lethargy, and seizures (Goel et al., 2023). Notably, among neonatal meningitis patients, infections mostly occur in premature infants, and they have a relatively high risk of developing neurological sequelae such as hydrocephalus after infection, indicating the severity and intractability of EM infection (Goel et al., 2023).

In adult patients, almost all infections are hospital-acquired, and the clinical manifestations are more diverse. Common manifestations include fever, dyspnea, and disturbance of consciousness. Some cases may be accompanied by skin and soft tissue infections, suggesting that the bacterium can invade through multiple routes and cause systemic inflammatory response (Umair and Nasir, 2021; Li et al., 2022). After immunocompromised patients are infected with this bacterium, the incidence rates of sepsis and respiratory tract infections are relatively high. Due to its multi-drug resistance, the treatment is difficult and the fatality rate is high (Umair and Nasir, 2021; Ma et al., 2023; Ferreira et al., 2024).

Although the clinical manifestations of EM infection are diverse, neonatal meningitis and sepsis and pulmonary infections in adult immunocompromised patients are the main manifestations. This prompts clinicians to remain vigilant in high-risk populations, make timely diagnoses, and take effective diagnostic and treatment measures to reduce mortality and the incidence of complications.

## 3 Diagnostic methods for *Elizabethkingia meningoseptica*

Due to the lack of specificity in the clinical manifestations of EM infection and the limitations of traditional detection methods, early and accurate diagnosis of EM infection faces challenges. Currently, a comprehensive diagnostic strategy is advocated, which combines clinical suspected diagnosis, microbiological confirmation, molecular precise identification, and complication evaluation.

For high-risk individuals presenting with fever of unknown origin, altered consciousness, respiratory failure, or neurological symptoms, the possibility of EM infection should be alerted (Li et al., 2022; Anita Wijaya et al., 2023). Initial laboratory tests often reveal an elevated white blood cell count and an increased proportion of neutrophils, accompanied by a significantly elevated C-reactive protein level, which reflects the body’s inflammatory state and the active phase of infection (Almatari et al., 2022; Anita Wijaya et al., 2023; Wang et al., 2026). This serves as an important basis for initiating etiological examinations (Anita Wijaya et al., 2023; Wu et al., 2023).

The diagnosis of EM infection mainly relies on microbiological culture. In particular, the culture of sterile body fluids such as blood and cerebrospinal fluid remains the “gold standard” for diagnosis. After obtaining a pure culture, traditional biochemical identification often leads to misjudgment due to similar reactions with other species in the *Elizabethkingia* genus, such as *Elizabethkingia anopheles*, so further confirmation is required (Dunn, 2023). Although matrix-assisted laser desorption/ionization time-of-flight mass spectrometry (MALDI-TOF MS) can distinguish EM from *Elizabethkingia anopheles*, its accuracy is highly dependent on the database used. For instance, a study from Singapore found that among 79 retrospective blood isolates initially identified by MALDI-TOF as either *E. meningoseptica* (96.2%) or *E. miricola* (3.8%), subsequent 16S rRNA gene sequencing revealed that 78 (98.7%) were actually *E. anophelis*, with only one true *E. meningoseptica* isolate (Chew et al., 2018). This highlights the risk of misidentification when using default MALDI-TOF databases. Consequently, the sequencing method based on the whole genome or housekeeping genes is regarded as the gold standard for species identification due to its high resolution and can effectively distinguish different strains within this genus (Lakicevic et al., 2023; Shropshire et al., 2025). In addition, with its simplicity, rapidity, and high throughput, the polymerase chain reaction is also an effective means for strain identification (van Belkum et al., 1995). These molecular techniques are not only used for identification, but also provide typing results that can be leveraged for traceability investigations during nosocomial outbreaks (van Belkum et al., 1995; O’Sullivan, 2014; Lau et al., 2021).

For patients suspected of having central nervous system infection or pneumonia caused by EM, imaging examinations should be promptly performed. The head imaging of meningitis may show characteristic changes such as ventricular dilation, hydrocephalus, meningeal enhancement, or the formation of brain abscesses, which is helpful for assessing the disease progression, guiding neurosurgical intervention, and predicting the prognosis (Barnawi et al., 2020; Goel et al., 2023). Chest imaging can reveal manifestations such as pneumonia consolidation and exudation, and evaluate the scope of pulmonary infection.

In conclusion, the diagnosis of EM infection is a multi-step process. It begins with clinical vigilance and screening of inflammatory indicators in high-risk patients, relies on the culture and isolation of sterile specimens, achieves accurate identification through molecular biology techniques, and finally determines the infection site and complications in combination with imaging. Establishing this comprehensive diagnostic pathway is the key to avoid misdiagnosis and delay and ultimately improve the prognosis of patients (Barnawi et al., 2020; Goel et al., 2023; Wu et al., 2023).

## 4 Resistance mechanisms and treatment of *Elizabethkingia meningoseptica*

### 4.1 Mechanisms of multidrug resistance

The multi-drug resistance of EM poses a central challenge in its clinical treatment. This resistance stems from a variety of intrinsic resistance genes on the chromosome and exogenous genes acquired through mobile genetic elements (MGEs), collectively resulting in a high level of resistance to the vast majority of commonly used antimicrobial agents.

The resistance mechanism of EM is primarily characterized by enzyme-mediated antibiotic inactivation. The chromosomes of EM commonly carry multiple β-lactamase genes, which form the basis for its intrinsic resistance to β-lactam antibiotics. Among them, the *bla*B and *bla*GOB genes encode class B metallo-β-lactamases (MBLs), which can efficiently hydrolyze carbapenems; the *bla*CME gene encodes an extended-spectrum β-lactamase, increasing the minimum inhibitory concentrations (MICs) of ceftazidime and cefepime and mediating EM resistance to cephalosporin antibiotics (Chen P. J. et al., 2024). Studies have shown that the combinations of these genes vary among different clinical isolates of EM, and this genetic diversity further exacerbates the complexity of drug resistance (Cai et al., 2025; Zhou et al., 2025). In addition, some EM strains also carry genes such as *ant* (6′)-Ib, which mediate resistance to aminoglycosides (Zhang et al., 2023). Moreover, some strains carry the *tet*(X) gene, and the fluorinated tetracycline monooxygenase encoded by this gene can lead to resistance to tetracyclines such as tigecycline, indicating further limitations in clinical treatment options (Yang et al., 2021; Morgado et al., 2024b).

Secondly, drug efflux mediated by efflux pumps is another key mechanism. The genome of EM encodes multiple efflux pump systems of the RND family (e.g., AdeFGH), which can actively expel various classes of drugs such as tetracyclines, fluoroquinolones, and chloramphenicol out of the cell. This enhances the strain’s tolerance to multiple antibacterial drugs and produces a synergistic effect with the enzyme-mediated mechanism, further exacerbating the treatment difficulty (Cai et al., 2025).

The spread of resistance genes is closely associated with various mobile genetic elements (MGEs), especially integrative and conjugative elements (ICEs). As a genetic element capable of transferring between bacterial chromosomes and plasmids, ICEs carry multiple resistance genes, including *tet*(X), which promotes the rapid spread of EM resistance genes. Studies have found that ICEs carrying *tet*(X) were detected in multiple EM genomes. These elements are highly conserved among different strains, suggesting that ICEs play a crucial role in the flow of EM resistance genes (Morgado et al., 2024b).

In summary, the multidrug resistance of EM is a multi-level comprehensive system. The broad-spectrum β-lactamases and efflux pumps encoded by inherent chromosomal genes form the backbone of drug resistance, while MGEs play a crucial role in the spread of drug-resistant genes. Its complex drug-resistance mechanism not only renders traditional antibiotic treatments ineffective but also suggests that future efforts should focus on strengthening molecular monitoring and implementing precision medication strategies to address the challenges in treating EM infections (Zhang et al., 2023; Morgado et al., 2024b; Cai et al., 2025).

### 4.2 Antibiotic susceptibility and treatment strategies

Currently, there are no globally standardized treatment guidelines for EM infections due to its rarity and complex resistance profiles, and therapy must therefore be guided by individual susceptibility testing results (Bennett and Blaser, 2014).

The multi-drug resistance of EM has led to extremely limited options for antimicrobial therapy. The development of effective anti-infection strategies must be based on accurate drug sensitivity testing and follow the principle of individualization.

Numerous studies have confirmed that EM can produce two types of β-lactamases: class A extended-spectrum β-lactamases and class B metallo-β-lactamases. These two enzymes enable EM to exhibit intrinsic resistance to the majority of β-lactam drugs, including penicillins, cephalosporins, and carbapenems (Ceyhan and Celik, 2011). However, the β-lactamase inhibitors sulbactam and tazobactam can significantly restore the sensitivity of EM to cefoperazone and piperacillin, respectively, resulting in a sensitivity rate of 88.5% for cefoperazone/sulbactam and 86.5% for piperacillin/tazobactam (Wang et al., 2020; Wu et al., 2023). However, the current novel β-lactamase inhibitor combinations, such as ceftazidime/avibactam and imipenem/relebactam, are ineffective against EM because these enzyme inhibitors primarily target class A, C, and D β-lactamases and are ineffective against the class B metallo-β-lactamases carried by EM (Kuo et al., 2021).

Currently, in addition to cefoperazone-sulbactam and piperacillin-tazobactam, drugs that still show a relatively high sensitivity rate in *in vitro* drug sensitivity tests include fluoroquinolones, minocycline, and trimethoprim-sulfamethoxazole (TMP/SMX). Due to their excellent pharmacokinetic properties and good penetration into the cerebrospinal fluid, fluoroquinolones were once considered as first-line drugs for the treatment of EM. However, due to mutations in the *gyrA* and *parC* genes, fluoroquinolone resistance rate has been increasing year by year, which limits their value of single application (Lin et al., 2019). Studies have shown that the 14-days mortality rate of patients with EM infection resistant to levofloxacin is significantly higher than that of sensitive patients (Huang et al., 2017). This result highlights the importance of early assessment of fluoroquinolone sensitivity to guide precise clinical medication. The susceptibility rate of minocycline to EM can reach 96% (Alhuthil et al., 2024). However, as it is a bacteriostatic agent, monotherapy may be ineffective in severe infections such as sepsis and meningitis, and its penetration in the central nervous system is limited. The susceptibility rate of trimethoprim-sulfamethoxazole is about 77% (Alhuthil et al., 2024), but its adverse reactions such as bone marrow suppression and nephrotoxicity limit its application in special patient populations. In addition, there are significant differences in the drug resistance rates of strains from different regions (Huang, 2024).

Given the aforementioned limitations, combination therapy has become a common strategy for treating severe EM infections, aiming to enhance efficacy, prevent drug resistance, and exert potential synergistic effects. Current combination therapies are mainly based on piperacillin/tazobactam, and drugs commonly used in combination with it include trimethoprim-sulfamethoxazole or fluoroquinolones (Zajmi et al., 2022). It must be clearly stated that vancomycin has no antibacterial activity against EM. However, in some previous individual case reports, its combination with ciprofloxacin, minocycline, or quinolones was effective, possibly due to unconventional drug synergy or other factors. This regimen lacks evidence-based support and should not be used as a routine choice (Barnawi et al., 2020; Spencer et al., 2020; Hashmi et al., 2023). Meanwhile, combinations that may produce antagonistic effects should also be avoided. For example, the combination of imipenem and vancomycin will reduce the therapeutic effect of the drugs (Yang et al., 2024). Any treatment regimen must be based on reliable drug susceptibility test results and comprehensively consider individual factors such as the site of infection, the patient’s liver and kidney functions, and immune status.

Patients infected with EM are often in critical condition. Active supportive treatment and complication management are the foundation for successful anti-infection, including systemic support such as maintaining hemodynamic stability, respiratory function support, and electrolyte balance. For patients with meningitis, intracranial pressure needs to be closely monitored, and neurological complications such as hydrocephalus and epilepsy should be promptly treated. In clinical cases, timely supportive treatment combined with rational use of antibacterial agents can significantly improve patients’ prognosis and reduce mortality (Ganesan and Sundaramurthy, 2022; Pelaez et al., 2025). Meanwhile, all treatments must be carried out in coordination under strict infection control measures to cut off the transmission chain and prevent recurrence or nosocomial infection outbreaks (Erinmez et al., 2021; Wu et al., 2023; Holcomb et al., 2024).

In conclusion, the extensive resistance of EM to traditional antibacterial agents poses a major obstacle to clinical treatment. Currently, cefoperazone-sulbactam, piperacillin-tazobactam, minocycline, compound sulfamethoxazole, and fluoroquinolones still retain activity against some strains. However, their clinical application must be strictly based on the drug sensitivity results, and the specific conditions of patients should be comprehensively considered. In the future, the development of new antibacterial agents and the optimization of combination therapy regimens need to be verified by more clinical studies to break through the current treatment dilemma (Kanashenko et al., 2021; Alhuthil et al., 2024; Alsayed et al., 2025). In addition, in-depth molecular analysis of the resistance mechanism will provide a scientific basis for achieving precise drug use and reducing the treatment failure rate. The successful management of EM infection requires the collaboration of multiple aspects. On the basis of rational anti-infection treatment, organ function support and the management of complications must be fully emphasized, and infection prevention and control measures must be strictly implemented to effectively improve the prognosis of patients and reduce the mortality rate.

## 5 Genomic research on *Elizabethkingia meningoseptica* and the spread of drug-resistant genes

### 5.1 Genomic characteristics

Genomic research on EM provides crucial insights into its pathogenicity, drug resistance mechanisms, and evolutionary relationships with other *Elizabethkingia* species at the molecular level (Lin et al., 2019; Hu et al., 2023).

The genome size of EM is generally around 3.9 Mb (Sun et al., 2015; Yikai et al., 2021), with a GC content of approximately 36.8%–37.0%, which is within the typical range of the Flavobacteriaceae family (Chen et al., 2016; Zhou et al., 2025). Genome analysis of EM has revealed a substantial number of predicted open reading frames (ORFs). For example, 7593 ORFs were identified in the type strain G4076, reflecting extensive genomic coding capacity (Girdhar et al., 2022). In contrast, the actual number of validated protein-coding genes in EM genomes is typically fewer than 4,000, as documented in publicly available genomes. For instance, strain Em3 contains 3673 protein-coding genes (Chen et al., 2017), while strain ATCC 21757 contains 3425 protein-coding genes, underscoring the distinction between predicted ORFs and functional genes.

Phylogenetic analyses have shown that EM and other *Elizabethkingia* species share varying degrees of genomic similarity, reflecting their evolutionary divergence (Liang et al., 2019). Multiple comparative genomic studies have indicated that EM and *Elizabethkingia anophelis* exhibit a high degree of similarity in their core genomes and functional gene clusters (Teo et al., 2014; Liang et al., 2019; Yang et al., 2021; Girdhar et al., 2022; Hu et al., 2022), despite their distant phylogenetic positions within the genus (Nicholson et al., 2018). This suggests that they may share many fundamental pathogenic mechanisms (Teo et al., 2014; Liang et al., 2019; Yang et al., 2021). However, comparative analyses of accessory genomes such as resistance genomic islands and virulence factors are more conducive to clarifying the epidemiological characteristics of the differences in niche selection and resistance profiles between the two species (Breurec et al., 2016; Lin et al., 2018; Andriyanov et al., 2024).

Functional annotation analysis indicates that the core genome of EM is rich in genes related to basic metabolism, such as those involved in pathways like the tricarboxylic acid cycle, oxidative phosphorylation, and vitamin K2 synthesis, which ensure its basic life activities and environmental adaptability (Yang et al., 2023). Beyond these housekeeping functions, the core genome also harbors conserved virulence determinants that contribute to its pathogenic potential (Chen et al., 2019). These include sialic acid transporters (neuC1 and neuC2) involved in immune evasion by mimicking host cell surface molecules, and curli synthesis genes that promote biofilm formation and adhesion to animal cells (Chen et al., 2017, 2019). EM additionally encodes glycoside hydrolases (GH) and polysaccharide utilization loci (PULs), including a GH33 sialidase (nanH) and an additional GH13 protein, which enhance metabolic versatility and environmental adaptability (Chen et al., 2019; Cao et al., 2023; Bax and Jurak, 2024). Furthermore, the core genome contains multiple two-component system proteins, transcription factors, and DNA-binding proteins that enable adaptive responses to environmental stimuli and regulate virulence gene expression (Agrawal et al., 2019; Chen et al., 2019). In contrast, its accessory genome exhibits higher plasticity and is rich in genes related to information processing and signal transduction (Zhong et al., 2019; Yang et al., 2021; Zhou et al., 2025). This “stable core + variable periphery” structure serves as an important genetic basis for EM to adapt to diverse environments, ranging from natural settings to hospital niches (Arredondo-Alonso et al., 2019).

The genomic basis of the multidrug-resistant phenotype of EM lies in the integration of a rich set of resistance genes on its chromosome, including the *bla*B gene encoding β-lactamase, the *bla*GOB and *bla*CME genes, the *aad*S gene mediating aminoglycoside resistance, and the *catB* gene mediating chloramphenicol resistance, etc., (Morgado et al., 2024b; Zhou et al., 2025). The multidrug resistance of EM not only stems from the inherent resistance genes on its chromosome but also because it can widely acquire and spread exogenous resistance genes through horizontal gene transfer (González and Vila, 2012; Morgado et al., 2024b). Among them, the conjugative transfer mediated by ICE plays a key role (Kaufman et al., 2020; Fu et al., 2021). ICE can not only integrate into the chromosome but also transfer between different strains through conjugation, efficiently disseminating the resistance genes it carries, such as *bla*B and *tet*(X) (Cury et al., 2017; Kaufman et al., 2020; Fu et al., 2021; Morgado et al., 2024b). This highly efficient horizontal spread ability is an important reason for the clonal outbreak and rapid evolution of drug resistance caused by EM in the hospital environment (Burnard et al., 2020; Fu et al., 2021).

In summary, the genome of EM exhibits the co-existence of a “stable core” and a “plastic periphery” (Chen et al., 2025). The core genome integrates essential metabolic genes, shared virulence determinants including sialic acid metabolism, biofilm formation and regulatory systems, and intrinsic resistance genes (Chen et al., 2017, 2019; Agrawal et al., 2019; Cao et al., 2023; Bax and Jurak, 2024). The extensive repertoire of resistance genes, often associated with mobile genetic elements as part of the genomic plasticity, is discussed in detail in Section “5.2 Distribution and spread of antibiotic resistance genes.” Given its high homology with *Elizabethkingia anopheles*, traditional biochemical identification faces challenges (Takei et al., 2025). The large reservoir of resistance genes in EM is often associated with mobile genetic elements, which forms the molecular basis for multidrug resistance and nosocomial transmission. Meanwhile, the abundant metabolic and adaptive genes in EM support its persistent colonization and spread in the hospital environment (Yang et al., 2021; Girdhar et al., 2022; Morgado et al., 2024b; Zhou et al., 2025). A comprehensive analysis of these genomic features is an important prerequisite for the development of precise diagnostic tools and novel intervention strategies.

### 5.2 Distribution and spread of antibiotic resistance genes

The continuous evolution and dissemination of EM multi-drug resistance are mainly attributed to its ability to extensively acquire and exchange resistance genes through horizontal gene transfer (Davin-Regli and Pagès, 2015; Evans et al., 2020). This process is primarily mediated by MGEs (Evans et al., 2020; Dimitriu, 2022).

A diverse array of MGEs contributes to resistance dissemination in EM, including integrative and conjugative elements (ICEs), insertion sequences (IS elements), transposons, and integrons (Bulagonda et al., 2018; Morgado et al., 2024b). However, the prevalence and significance of these elements vary substantially. Genomic analyses of hundreds of EM isolates have revealed that ICEs are the most prominent and well-characterized MGEs, while plasmids appear to play a negligible role, with only two plasmids identified across 202 *Elizabethkingia* genomes, neither associated with resistance genes (Jin et al., 2024).

Comparative genomic analysis based on whole-genome sequencing (WGS) reveals that numerous MGEs, including plasmids, integrons, transposons and ICEs, are present in the genomes of EM and its closely related species such as *Elizabethkingia anopheles* (Xu et al., 2018). These elements carry and transfer various antibiotic resistance genes, such as those encoding β-lactamases, aminoglycoside-modifying enzymes, and trimethoprim resistance genes (Yang et al., 2021; Zhou et al., 2025). The differences in MGEs among different strains directly shape the strain-specific antibiotic resistance profiles and dynamically reflect the flow and recombination of the antibiotic resistance gene pool within the population (Tran et al., 2026).

Among numerous mobile genetic elements (MGEs), integrative and conjugative elements (ICEs) have greatly promoted the spread of EM resistance genes due to their characteristics of chromosomal integration stability and high-efficiency conjugative transfer (Osorio et al., 2008). ICE-mediated conjugation relies on a type IV secretion system (T4SS), which forms a pilus and translocation channel for DNA transfer between donor and recipient cells (Leonetti et al., 2015; Bulagonda et al., 2018; Kohler et al., 2019). Represented by ICEEmSQ101, this type of ICE is approximately 49,769 bp in length and harbors multiple resistance genes, including *tet*(X) and aadS (Morgado et al., 2024b). These genes can be transferred to recipient bacteria simultaneously during a single conjugation event, significantly accelerating the formation and spread of multidrug - resistant phenotypes (Morgado et al., 2024b). Beyond ICEEmSQ101, other ICEs carrying diverse resistance gene arrays have been identified in EM and related species, including elements capable of transferring β-lactamase genes (*bla*B, *bla*GOB, *bla*CME) and efflux pump genes (Breurec et al., 2016).

The host range of ICE-mediated HGT extends beyond EM itself. Evidence indicates that ICEs similar to those in EM can transfer to other *Elizabethkingia* species and even to phylogenetically related genera such as *Chryseobacterium* within the family Flavobacteriaceae (Fu et al., 2021). For example, the ICE ICECspPOL2 originally identified in *Chryseobacterium* has been shown to transfer horizontally to *Elizabethkingia* species, highlighting the potential for inter-genus dissemination of resistance determinants (Fu et al., 2021).

Molecular epidemiological investigations of ICEEmSQ101 have revealed that its distribution in China exhibits regional clustering characteristics, strongly suggesting that the spread of resistance genes is not random but is jointly driven by local antibiotic use pressure, specific medical practices, and the prevalence of dominant clonal strains (Wang et al., 2023, 2024). On one hand, this clustering indicates that the spread may be geographically limited at present. On the other hand, it also warns that once a dominant resistant clone is established in a region, it can easily trigger infection outbreaks in hospitals or even in the community (Baker et al., 2018; Morgado et al., 2024b; Rahbé et al., 2024).

From a clinical perspective, EM strains carrying ICEEmSQ101 pose a significant challenge to antibiotic treatment due to their extensive spectrum of resistance genes (Cao et al., 2017), rendering simple drug - susceptibility testing insufficient for effective management. Therefore, molecular epidemiological investigations of EM and its carriage of resistance genes must be strengthened. Active screening of resistance elements based on genomics should be carried out in high-incidence areas and key departments. WGS technology should be utilized to precisely trace the clonal transmission chains and the transfer pathways of MGEs during outbreaks (Matsumura et al., 2025). Subsequently, combined with the characteristics of locally prevalent resistance elements, it can guide empirical clinical medication and optimize hospital infection control measures to prevent the spread and outbreaks of resistant strains (Morgado et al., 2024b; Cai et al., 2025).

In conclusion, the distribution and spread of EM resistance genes are highly dependent on mobile genetic elements, particularly ICEs, while plasmids play a minimal role. The ability of ICEs to transfer across species and genus boundaries underscores the need for surveillance beyond traditional pathogen-centric approaches. Taking ICEEmSQ101 as an example, it, as a “resistance gene complex,” achieves efficient horizontal spread and exhibits epidemiological characteristics of regional aggregation. This requires that future prevention and control strategies must be elevated from “targeting bacteria” to “targeting resistance genes.” Molecular monitoring should be used to understand the spread dynamics, providing a key entry point for blocking the spread of drug resistance.

### 5.3 Genomic insights into clinical diagnosis and drug development

Genomics, as a crucial tool, is profoundly transforming the clinical practice of EM infections. Its most direct implication lies in guiding precise diagnosis and personalized treatment. Accurate species identification is the foundation of appropriate therapy, however, conventional methods often misidentify *Elizabethkingia anophelis* as EM due to database limitations, with one study reporting a correct identification rate of only 24.5% for automated systems (Rahim and Gupta, 2019). This misdiagnosis carries clinical risk, as the two species exhibit differences in antimicrobial susceptibility, for instance, EM is less susceptible to piperacillin-tazobactam and levofloxacin than *E. anopheles* (Lin et al., 2018). Therefore, empirical regimens intended for EM may therefore be ineffective for *E. anophelis* infections.

Empirical therapy for EM itself is also challenging, with mortality rates ranging from 23.4% to 65.6% in published case series, particularly in immunocompromised patients or those with central venous catheters (Govindaswamy et al., 2018; Ma et al., 2023). The intrinsic resistance of EM to most β-lactams, carbapenems, and aminoglycosides, mediated by chromosomal metallo-β-lactamases (*bla*B, *bla*GOB) and extended-spectrum β-lactamases (*bla*CME), renders many empirical choices ineffective (Hem et al., 2022; Morgado et al., 2024a; Wu et al., 2024). WGS addresses these challenges by enabling comprehensive detection of resistance determinants. For example, WGS has revealed that EM strains carry resistance genes such as *aadS* (aminoglycoside resistance) and *catB* (chloramphenicol resistance), which would not be predicted by conventional methods (Morgado et al., 2024b; Wu et al., 2024).

Compared with the time-consuming traditional drug susceptibility tests, WGS or targeted sequencing can systematically identify all known drug-resistant genes in a short period, achieving a leap from “phenotypic drug susceptibility” to “genotypic prediction.” This provides a precise basis for clinicians to select effective antibiotics and avoids treatment failure caused by blind medication. Each approach has distinct advantages and limitations. Phenotypic testing directly measures bacterial growth inhibition and provides quantitative MIC values essential for dosing decisions, but it is time-consuming. Genotypic prediction offers rapid turnaround and can detect resistance genes directly from clinical samples without culture, but it is expensive, requires specialized expertise, and cannot determine whether detected genes are functionally expressed or provide MIC data. Therefore, an integrated approach combining rapid genotypic screening with confirmatory phenotypic testing may represent the optimal strategy for managing EM infections.

Beyond guiding diagnosis and treatment selection, genomic analysis has also illuminated fundamental biological features of EM that can be exploited for therapeutic development. Genomics has also revealed, several metabolic pathways in EM that are distinct from human hosts, providing potential targets for new drug design. Through in-depth analysis of the whole genome of this bacterium, researchers have identified a number of species-specific or genus-specific metabolic features that are essential for bacterial survival and absent in humans, making them attractive candidates for antimicrobial development (Chen et al., 2019; Kanashenko et al., 2021).

One well-characterized example is the vitamin K2 (menaquinone) biosynthesis pathway, which serves as an electron carrier in the bacterial respiratory chain (Yang et al., 2023). This pathway involves key enzymes encoded by the menA, menD, menH, and menI genes, which have shown higher expression in certain EM strains and are essential for bacterial viability (Kanashenko et al., 2021). Notably, the menD gene catalyzes the first committed step in menaquinone biosynthesis and is subject to feedback inhibition by 1,4-dihydroxy-2-napthoic acid (DHNA), with specific arginine residues (Arg97, Arg277, Arg303) critical for both enzyme activity and regulation (Bashiri et al., 2019). Unlike bacteria, humans cannot synthesize vitamin K2 and must acquire it through diet, making this pathway a promising target for selective antimicrobial drugs (Kanashenko et al., 2021).

Another unique metabolic feature of EM is its sialic acid metabolism pathway. EM strains carry genes involved in sialic acid synthesis (nagB, glmS, glmB), transport (neuC1, neuC2), and degradation (nanH) (Vann et al., 2004; Gunawan et al., 2005; Chen et al., 2019). Notably, neuC1, neuC2, and nanH are present in EM but absent in other *Elizabethkingia* species such as *E. anophelis* and *E. miricola*, suggesting species-specific pathogenic mechanisms (Chen et al., 2019). Sialic acids play critical roles in immune evasion by allowing bacteria to mimic host cell surface molecules, and they can also serve as carbon and nitrogen sources when environmental nutrients are scarce (Chen et al., 2019). This dual role in pathogenesis and nutrient acquisition makes sialic acid metabolism an attractive target for therapeutic intervention (Agrawal et al., 2019).

*Elizabethkingia meningoseptica* also possesses an extensive array of carbohydrate-active enzymes (CAZymes) and polysaccharide utilization loci (PULs), which contribute to its metabolic versatility and environmental adaptability (Chen et al., 2019). CAZyme-encoding genes constitute approximately 3% of the EM genome, including up to 58 glycoside hydrolases (GHs) (Chen et al., 2019). Notably, EM specifically encodes a GH33 sialidase and an additional GH13 protein, which are features not found in other *Elizabethkingia* species (Chen S. et al., 2024). The bacterium contains 25 loci encoding SusC-SusD homologs, with a specific PUL encoding a GH30_3 enzyme (β-1,6-glucanase) that is unique to EM (Chen et al., 2019; Li et al., 2023). These specialized metabolic capabilities enable EM to utilize diverse carbon sources (e.g., D-maltose, D-trehalose, D-glucose, D-mannose), form biofilms on medical surfaces, and persist in hospital environments (Chen et al., 2019). Disrupting these pathways could compromise the bacterium’s ability to acquire nutrients and colonize host tissues.

The discovery of such unique metabolic pathways and protein targets, including menaquinone biosynthesis, sialic acid metabolism, and specialized carbohydrate utilization systems, provides new insights for the development of antibacterial drugs. Particularly in the context of the increasingly severe resistance to traditional antibiotics, novel drugs targeting pathogen specific metabolic pathways have important clinical application value (Girdhar et al., 2022).

In addition, genomics is revolutionizing the hospital infection monitoring and outbreak early-warning systems. Monitoring the population plasticity of EM and the transmission ability of mobile genetic elements requires the upgrading of infection control to the molecular level. Molecular typing and source-tracing technologies based on WGS can accurately identify outbreak clones, trace transmission routes, and monitor the diffusion dynamics of high-risk drug-resistant clones in real-time, thus transforming the prevention and control mode from passive post-event response to active pre-event early warning (Morgado et al., 2024b; Zhou et al., 2025).

In summary, genomics is comprehensively reshaping strategies for combating EM infections, including facilitating rapid molecular diagnosis, guiding new drug development, and enabling precise infection control. In the future, promoting the in - depth integration of clinical microbiology, bioinformatics, and diagnosis - treatment systems and constructing genome-phenotype association maps will be the key to achieving precision medicine in the true sense (Girdhar et al., 2022; Morgado et al., 2024b; Zhou et al., 2025).

## 6 Hospital infection control and prevention measures

### 6.1 Sources of infection and routes of transmission

As an important nosocomial pathogen, EM has formed a clear epidemiological chain regarding its infection sources and transmission routes.

The water supply system in hospitals serves as a crucial environmental reservoir. EM can spread through hospital water systems, especially by forming biofilms within pipes. This enables bacteria to survive and reproduce in the water source, becoming a concealed source of nosocomial infections. Although research has indicated that in hospital water supply systems, low-level chlorine treatment (free chlorine concentration × contact time value < 0.04 mg⋅min/L) can achieve 99.9% inactivation of *Elizabethkingia* bacteria (Holcomb et al., 2024), bacteria inside biofilms are physically protected by the matrix and in a state of metabolic slowdown. As a result, their resistance to disinfectants significantly increases. This makes it difficult for conventional water treatment methods to completely remove the formed biofilms, causing the water continuously contaminated by biofilms to become a long-term and concealed source of infection.

Contaminated medical equipment serves as a crucial transmission medium. EM can contaminate devices such as ventilator humidification tanks and nebulizers. Subsequently, pathogens can infect patients through two routes: contact transmission via contaminated hands or instruments, and aerosol transmission (Guchhait et al., 2023; Alhuthil et al., 2024). Notably, the transmission of EM is not limited to the hospital environment but is also related to its distribution in nature. For instance, studies have found an association between EM and the gut microbiota of *Anopheles* mosquitoes, suggesting that it may be indirectly transmitted through vectors such as insects (Egyirifa and Akorli, 2024). Aquatic animals like bullfrogs can also carry EM, broadening its ecological niche and complicating efforts to control its entry into healthcare environments, even though direct transmission from these animals to humans has not been demonstrated (Tsai et al., 2023).

In conclusion, the in-hospital transmission of EM is a continuous process that starts from environmental colonization, progresses to device contamination, and then affects high-risk patients through specific routes. A profound understanding of this complete chain is the cornerstone for formulating targeted prevention and control strategies.

### 6.2 Infection control strategies

The foundation of preventing EM infections lies in strictly implementing aseptic operating procedures and regularly disinfecting medical equipment and the hospital environment. EM infections are more common in patients receiving invasive procedures such as mechanical ventilation and central venous catheterization. Given its inherent multi-drug resistance, prevention is particularly important, with the core being to break the transmission route (Erinmez et al., 2021; Goel et al., 2023). Specifically, strict aseptic operations, including standardized hand hygiene, the use of sterile gloves, thorough disinfection of the puncture site, and standardized catheter insertion and maintenance procedures, can effectively reduce the risk of cross-transmission. On this basis, implementing targeted source control and strengthening environmental and equipment management is crucial.

Given EM’s ability to form biofilms in water systems, conventional disinfection is often inadequate (Vertes et al., 2012). Biofilm-embedded EM cells can require significantly higher disinfectant doses or longer contact times than planktonic cells, up to 600-fold for chlorine (Holcomb et al., 2024). For hospital water systems, free chlorine at 0.2 mg/L residual achieves 99.999% inactivation of planktonic EM within 1 min (CT value < 0.04 mg⋅min/L) (Holcomb et al., 2024). However, to control biofilms, more aggressive measures such as shock chlorination may be necessary (Assaidi et al., 2020). Installing point-of-use filters on taps and showers in high-risk units may effectively prevent exposure to waterborne EM (Camps et al., 2011). Regular inspection and annual chlorination of hospital water tanks are recommended, along with routine microbiological monitoring of water outlets and biofilms (Pawar et al., 2016; Kottapalli et al., 2021).

For dental unit waterlines, effective protocols include continuous use of 0.02% hydrogen peroxide combined with weekly shock treatments of 0.25% hydrogen peroxide, overnight treatment with 0.12% chlorhexidine and 12% ethanol, or overnight application of a 1:50 dilution of Listerine (Pawar et al., 2016). For medical equipment and environmental surfaces, chlorine-based disinfectants such as sodium hypochlorite with thorough scrubbing are recommended (Marchand et al., 2017; Assaidi et al., 2020). Chlorine dioxide at 120–200 ppm has demonstrated >99.99% reduction of biofilms within 30 s to 5 min (Norville et al., 2025). Peracetic acid alone or combined with hydrogen peroxide are also effective options (Goetz et al., 2022).

Given that biofilm-embedded EM cells can require up to 600 times higher disinfectant concentrations than planktonic cells (Holcomb et al., 2024), the efficacy of disinfectants should be validated against biofilm-associated organisms. Regular audits of cleaning practices and environmental surveillance cultures are essential for sustained control.

Considering the characteristic of EM to form biofilms in water systems, a special maintenance plan for water systems should be formulated, including regular water quality monitoring and microbial testing, and enhanced disinfection measures should be taken for colonized systems (Guchhait et al., 2023; Holcomb et al., 2024). Meanwhile, it is essential to ensure that medical equipment related to water sources or aerosols undergoes strict high-level disinfection or sterilization, and effective daily cleaning and disinfection of environmental surfaces should be carried out (Guchhait et al., 2023).

At the clinical management level, for patients with multi-drug-resistant bacterial infections confirmed or colonized with EM, it is recommended to implement contact isolation measures to reduce the risk of cross-transmission (Ferreira et al., 2024). Meanwhile, optimizing and restricting the overuse of broad-spectrum antibiotics such as carbapenems through the hospital antimicrobial stewardship program, so as to reduce the selective pressure on resistant bacteria such as EM, is a key step in fundamentally preventing their spread.

Finally, establishing an active monitoring and outbreak response mechanism is the core of improving the closed-loop prevention and control. It is recommended to conduct active microbial screening for high-risk patients in high-risk departments such as neonatal intensive care units to achieve early detection. Once a suspected infection cluster or outbreak occurs, an epidemiological investigation should be initiated immediately, and molecular typing techniques should be used for accurate source tracing to identify the transmission chain and guide the implementation of precise intervention measures (Morgado et al., 2024b; Zhou et al., 2025).

The effective control of EM infection relies on a multi-level system: taking standard precautions as the cornerstone, water system and equipment management as the key breakthrough point, isolation and antimicrobial stewardship as supplements, and active surveillance and molecular source - tracing as early-warning and evaluation tools. This is the fundamental way to systematically reduce the risk of its infection.

## 7 Conclusion

As a nosocomial pathogen with complex drug resistance, EM poses a severe threat to neonates and immunocompromised patients. This review systematically summarizes the epidemiological characteristics, clinical manifestations, and diagnostic difficulties of EM infections, and delves into its multiple drug resistance mechanisms, including chromosomally encoded broad-spectrum β-lactamases, active efflux pumps, and horizontal gene transfer mediated by integrative conjugative elements. These mechanisms collectively lead to extensive drug resistance, presenting formidable challenges to clinical treatment.

Currently, effective management of EM infections hinges on the coordinated implementation of two key strategies. On one hand, rapid and accurate diagnosis is the prerequisite for effective treatment, which is mainly achieved through the application of molecular diagnostics and genomic sequencing technologies. This promotes the shift of clinical practice from empirical medication to personalized treatment based on clear information about drug-resistant genes. On the other hand, strict infection control is fundamental for blocking transmission. Core measures include managing hospital water systems and removing biofilms, thoroughly disinfecting and sterilizing medical devices, and implementing standardized contact isolation for high-risk patients who are infected or colonized. These two strategies support each other and together form the practical basis for curbing pathogen transmission and improving patients’ clinical outcomes.

Looking ahead, the key to solving the problem of EM infection lies in promoting the in-depth integration of cutting-edge genomic research and new drug development practices. By means of technologies such as high-throughput sequencing, clarifying its drug-resistance mechanism and evolutionary rules will provide theoretical guidance for targeted drug design. Meanwhile, actively developing new antibacterial drugs and exploring reasonable combination drug regimens are expected to overcome the existing drug-resistance barriers, thereby reducing the infection-related mortality and the risk of complications.

In summary, the prevention and treatment of EM infections require the integration of precise diagnosis, personalized treatment, and strict infection prevention and control measures. The ultimate goal is to effectively manage and control EM infections and improve patients’ prognosis.
